# Heritability of Morphology in Brook Trout with Variable Life Histories

**DOI:** 10.1371/journal.pone.0012950

**Published:** 2010-09-23

**Authors:** Anna Varian, Krista M. Nichols

**Affiliations:** 1 Department of Forestry and Natural Resources, Purdue University, West Lafayette, Indiana, United States of America; 2 Department of Biological Sciences, Purdue University, West Lafayette, Indiana, United States of America; University of Missouri, United States of America

## Abstract

Distinct morphological variation is often associated with variation in life histories within and among populations of both plants and animals. In this study, we examined the heritability of morphology in three hatchery strains of brook trout (*Salvelinus fontinalis*), which were historically or are currently used for stocking and supplementation of both migratory and resident ecotypes in the upper Great Lakes region. In a common garden experiment, significant variation in body morphology was observed within and across populations sampled at three time periods. The most notable differences among strains were differences in dorso-ventral body depth and the shape of the caudal peduncle, with some differences in the anterior-posterior placement of the dorsal and ventral fins. Variation with and among 70 half-sib families indicates that heritabilities of morphology and body size were significant at most developmental time points both within and across strains. Heritabilities for morphological characters within strains ranged from 0 to 0.95 across time points. Significant within-strain heritabilities for length ranged from 0 to 0.93 across time points and for weight ranged from 0 to 0.88. Significant additive genetic variation exists within and across hatchery brook trout strains for morphology and size, indicating that these traits are capable of responding to natural or artificial selection.

## Introduction

Morphological features are some of the most obvious traits associated with adaptation and life history diversity in all organisms, including fishes. For example, morphological features such as body armor in threespine sticklebacks (*Gasterosteus aculatus*) are related to differential predation across habitat types [1]. In cichlid fishes, jaw morphology is related to functional feeding ecology [2], [3]. In salmonid fishes, whole body morphology is tightly associated with variability in migratory and resident life histories [4], [5], [6]. In all, it is clear that morphological features are associated with the ecology of organisms, and have evolved or diversified within and among populations and species. In some of the above examples, decades of studies have indicated that both genetics and environment play a role in the phenotypic diversity observed in nature; however, for many non-model fish species, the question of whether genetics, environment, or genotype-by-environment interaction (or a combination of these effects) shapes phenotypic diversity remains to be answered.

Phenotypic diversity of body shape is one of the most obvious differences among members of the same species. Specialized body shapes have been found to reflect ecological adaptations to habitat, life history, food resources, and the presence of predators [1], [5], [6], [7], [8], [9], [10], [11], [12], [13], [14], [15], [16]. Fish migrating between fluvial and open-water habitats experience environments that select for different morphological optimums. For example, in preparation for migration, anadromous salmonids undergo morphological changes [17], [18], [19]. These morphological changes are associated with long distance, sustained swimming during migration, and include features such as a narrow caudal peduncle and streamlined body for minimizing drag [20]. Slight differences in morphology can result in variations in optimum swimming speed, the metabolic cost of swimming, and sustained swimming ability [21], [22], [23], [24].

Across their native range in North America, brook trout exhibit a variety of life history strategies, and these include individuals that are river resident for all of their lives (fluvial), migratory individuals that use both river and lake or ocean environments, and lake dwelling (lacustrine) ecotypes [25]. These life histories are similar to the well studied *Oncorhynchus* sps. in which morphology is known, in part, to be under genetic control and associated with life history traits [26], [27]. Inadvertent selection associated with the hatchery environment has raised concerns about the preservation of genetic variation in traits associated with fitness in the wilde populations. Fleming and Gross [28] suggested that hatchery strains should be more streamlined due to the loss of the need for burst swimming which is facilitated by a deep body. A number of studies [28], [29], [30] have found hatchery strains of *Oncorhynchus* sps to be more streamlined than their wild origins. In this study, we examine genetic variation in shape within and across hatchery strains of brook trout that are used to supplement populations with diverse life history strategies.

Morphological variation can be a product of both phenotypic plasticity across environments and heritable genetic variation. For example, in some fish species differentiation in body shape can be induced by water velocity [31], [32], [33]. Several studies have identified morphological differences between sympatric ecotypes of salmonids [4], [11], [12], [34], [35], [36], [37]; however, only a handful of studies in a limited number of species have determined if body morphology within and among populations of salmonids is heritable [5], [38], [39], [40], [41]. Though not explicit studies of heritability, Morinville and Rasmussen [4], [42] found populations of wild juvenile anadromous brook trout to be more streamlined with shorter paired fins and, on average, occupied faster current speeds then their fluvial counterparts. Whether or not these fish occupy faster currents because their morphology suits the environment or these fish are more streamlined as a result their environment is unknown.

Understanding whether phenotypic diversity in Great Lakes brook trout reflects underlying genetic variation for ecologically and evolutionarily important quantitative traits has important implications for conservation and management of extant populations. Prior to the 1990s, brook trout stocked into Lake Superior originated from hatchery strains derived from populations outside the basin without consideration for local adaptation [43]. However, population genetic studies suggest that these historical stockings were largely unsuccessful [44]. The lack of success in historical reintroduction attempts of migratory or lacustrine forms of brook trout may be attributed to the lack of a basic understanding of the biology of alternative ecotypes from different systems, as well as of the ecological conditions and evolutionary history that have shaped extant life history diversity [45], [46]. A current lack of understanding for the mechanisms promoting diversity in migration and residency in brook trout in the upper Great Lakes and the causes of long term population declines have lead both biologists and the public to pay special interest to brook trout conservation and reintroduction efforts in Lake Superior [45], [46], [47], [48]. However, to date, no studies on the heritability of characters associated with alternative life history strategies, either in hatchery or natural populations of upper Great Lakes brook trout, have been conducted.

In this study, we quantify genetic variation in morphological and size-related traits within and among hatchery brook trout strains. The strains chosen for study include brook trout historically or currently stocked into the upper Great Lakes region, and originated from both migratory and resident populations. By measuring narrow-sense heritability, we gain information about whether genetic variation contributes significantly to variation in the phenotype, and whether genetic variation is available for adaptation and evolution within these stocks. In this study, we report on the morphological characteristics in hatchery brook trout originating from three source populations, testing the null hypotheses that: 1) morphometrics of brook trout strains do not differ; 2) morphometric differentiation does not change over time; and 3) morphological variation exhibits no underlying additive genetic variation. Estimates of heritability for traits in wild populations are difficult to obtain; estimation or observation of the pedigree relationships of individuals, accounting for environmental parameters, and reliable field estimation of the phenotypes can be challenging and require extensive resources [49]. We conducted a common garden experiment, thus minimizing environmental contributions to any observed differences in phenotypes.

## Materials and Methods

### Ethics statement

Work with the fish in this study was approved by the Purdue Animal Use and Care Committee (protocol ID 06-051).

### Fish strains and crosses

Three strains of brook trout were used for morphometric analysis: Siskiwit, Assinica, and Iron River. Siskiwit brook trout are a migratory strain from the Big and Little Siskiwit Rivers on Isle Royale, Michigan [46]. The hatchery broodstock originated from 8 males and 11 females collected from these locales in 1995 and 1999. In a continuing effort to maintain the genetic structure of the natural populations the U.S. Fish and Wildlife Service collected additional gametes from a wild female and two males in 2004 (H. Quinlan, U.S. Fish and Wildlife Service and D. Bast, U.S. Fish and Wildlife Service personal communication). The gametes used in this study came from the U.S. Fish and Wildlife Service Iron River National Fish Hatchery (Iron River, Wisconsin). Assinica and Iron River strains were obtained from Michigan Department of Natural Resources, Marquette State Fish Hatchery (Marquette, Michigan). Both the Assinica and Iron River strains are currently stocked in lakes and streams within the Lake Superior basin [50]. The Assinica strain was founded from four females and three males collected in late summer near the outlet of Lake Assinica, Quebec, in 1962. These brook trout were presumably migrating from Lake Assinica downstream into the Broadback River to spawn [51], [52]. Iron River brook trout are a fluvial strain established from 1,400 fish collected in 1993 from the Iron River, Michigan [53]. Though additional hatchery strains are used for stocking efforts in Lake Superior and surrounding watersheds, we were unable to obtain gametes from the Tobin Harbor and Nipigon Bay hatchery strains (two strains that spend at least a portion of their lives in Lake Superior) at the same time gametes were available from the other strains used.

Full-sib nested half-sib and partial factorial mating designs were used to generate families with half-sibling relationships (sharing either a male or female parent) for estimation of heritability. In general, milt from one male was used to fertilize eggs from two or three females. However, in cases of high fecundity, egg lots were split and fertilized with more than one male to create more families. A total of 12 females (dams) and 5 males (sires) of the Siskiwit strain were chosen to create 12 families in which each sire was mated to 2 or 3 dams. Fifteen dams and 10 sires were used to create 30 Assinica families with each sire mated to 3 dams and each dam mated to 2 sires. Twenty dams and 10 sires were used to create 28 Iron River families with each sire mated to 2 or 3 dams and eggs of 8 dams were split and fertilized by 2 sires. Ten eggs from each female were measured with digital calipers to the nearest 0.01 to determine mean egg diameter.

Families were created on 09 and 15 November 2006. All fish were reared at Purdue University Aquaculture Research Laboratory (West Lafayette, Indiana) under the same laboratory conditions. Embryos were incubated in two Heath stack incubators sharing a recirculating system at 9.5±1.5°C. Development rate was measured in 80 embryos from each female as time from fertilization to hatch expressed in accumulated temperature units, as described by Robison et al. [54], to evaluate differences among strains in development rate. At swim-up, when the fry had utilized all yolk resources, full-sib families were moved and subsequently held in 19 L buckets modified with screen siding to allow water to flow through. These buckets were held within five 2,177 L flow-through circular tanks receiving well water (13.0±2.0°C), and families and strains were randomized among tanks. Fry were fed once daily to satiation with Bio-Oregon Bio-Vita trout feed. In April 2007, densities were equalized among families to 100 fish or less.

In June 2007, all fish were tagged according to family with Visible Implant Elastomer (Northwest Marine Technology, Inc., Shaw Island, Washington) and released from their buckets into the 2,177 liter tanks. A natural photoperiod corresponding to local conditions was maintained from swim-up throughout the study. Fish were fed daily with Bio-Oregon Bio-Vita trout feed at biomass percentages calculated according to average body weight across all tanks. After the fry stage, daily feed rates were adjusted at each sampling period according to current information on tank density and average fish weight.

### Morphometrics

In this study, we use geometric morphometric methods to quantify shape variation. A number of methods have been devised to analyze shape, but many fail to correctly remove size variation and may also inadvertently remove shape variation in their attempt [55], [56]. On the other hand, geometric morphometrics relies on geometry of all digitized landmarks together (rather than individual inter-landmark distances) to produce shape coordinates independent of size [57] and allows for body shape of individual specimens to be reconstructed in the form of clearly interpretable thin-plate splines, showing the deformation of form from the average shape and the covariation among digitized landmarks [58]. Geometric morphometric methods have been found to be more powerful in detecting slight differences between shapes, as would be expected within species [55], [58], [59]. For all of the reasons stated above, we chose to use geometric morphometrics to analyze shape variation among strains and for heritability.

Morphometric sampling took place on three separate occasions in the first two years of life: from 13–17 August 2007 (sampling period one), 22–26 October 2007 (sampling period two), and 21–24 January 2008 (sampling period three). Field studies have shown that brook trout on the south shore of Lake Superior migrate out to the lake during their second year of life; however, mass out-migrations of juveniles at any specific time of year have yet to be detected [60]. During sampling period one, 660 fish were sampled: 132 Siskiwit from 12 families, 256 Assinica from 27 families, and 257 Iron River from 20 families. During sampling period two, 496 fish were sampled: 66 Siskiwit from 12 families, 232 Assinica from 27 families, and 198 Iron River from 20 families. During sampling period three, 348 fish were sampled: 36 Siskiwit from 11 families, 183 Assinica from 26 families, and 129 Iron River from 20 families. With the exception of the total loss of a single family from Siskiwit, all families from each of the strains were sampled at each time point, with a decrease in the number of individuals sampled per family due to mortality or tag loss during the course of the study. Since individual identification was not possible with the tags only identifying families, it is possible that the subsample made from each family at each time point contained individuals previously sampled, but when family size exceeded our sample size, it is also possible that some individuals at each time point were not sampled before or in subsequent samplings. To avoid stomach bulge that could influence morphometrics, fish were not fed for 24 h prior to sampling. Fish were anesthetized with tricaine methanesulphonate (MS-222, Argent Chemicals, Redmond, WA), and total length (mm) and wet weight (g) measurements were taken. Digital photographs were taken of the left side of each fish as described by Nichols et al. [61]. After sampling, fish were returned to their original tanks. Because individual identification tags were not used, fish may or may not have been sampled again during the next sampling period.

**Figure 1:**
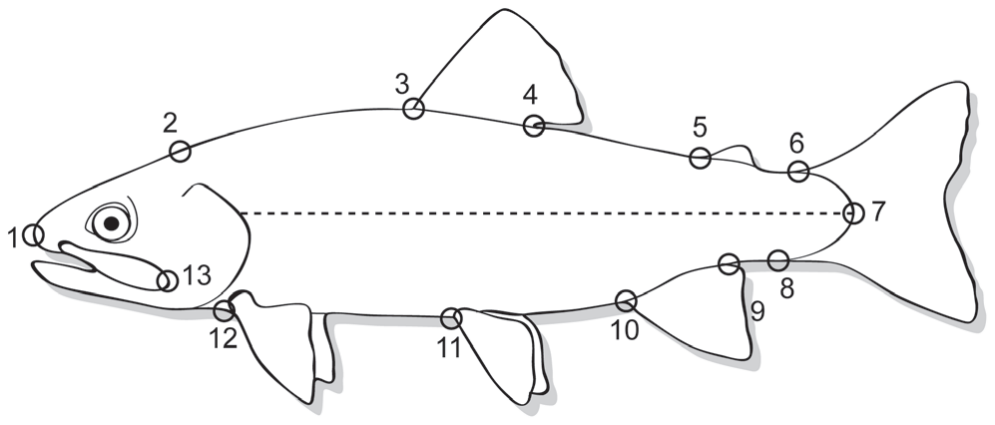
Location of the 13 landmarks used to describe morphometric variation in brook trout. (1) anterior tip of snout, (2) posterior aspect of neurocranium, (3) origin of dorsal fin (4) insertion of dorsal fin, (5) origin of adipose fin, (6) anterior attachment of dorsal membrane from caudal fin, (7) base of middle caudal rays, (8) anterior attachment of ventral membrane from caudal fin, (9) insertion of anal fin, (10) origin of anal fin, (11) origin of pelvic fin, (12) origin of pectoral fin, (13) posterior end of maxillary.

To evaluate body shape, 13 landmarks (Figure 1) after Winans [19] were digitized using tpsDig [62] from the tps software series (available at http://life.bio.sunysb.edu/morph/). To maintain consistency all points were digitized by a single individual. Landmark coordinates obtained were used in a relative warps analysis using the software tpsRelw [63]. Briefly, tpsRelw uses a generalized Procrustes analysis (GPA) to compute a consensus shape with a minimum sum of squared Procrustes distances to other specimens wherein each specimen is individually superimposed onto the average of previous specimens until all specimens are averaged. The GPA thus removes variation among samples due to location, orientation, and scale [63]. The consensus shape is used as a reference in which all specimens are compared [64]. The multivariate measure of size (centroid) of each specimen is computed as the square root of the sum of squared inter-landmark distances [65]. Eigenvectors of the bending energy matrix create shape variables, or partial warp scores, composed of both affine (uniform) and non-affine (non-uniform) shape variation for use in multivariate statistical analysis [65]. A singular value decomposition of partial warp scores yields relative warp scores. In addition to overall body shape, relative condition factor (K_n_) was calculated as:
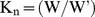
(1)where W is the weight (g) of an individual and W' is the standard weight for an individual of given length determined from the following weight-length regression calculated from all sampled brook trout [66]:
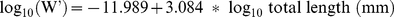
(2)Relative condition factor (K_n_) was used as it compensates for allometric growth and can be compared across all lengths, populations, and even different species [66].

### Statistical analyses

#### Summary statistics

To determine if differences in morphology exist among strains, a multivariate analysis of variance (MANOVA) was conducted on the partial warp scores at each sampling period. A multivariate analysis of covariance (MANCOVA) using log_10_ centroid size as a covariate was conducted to test for differences in allometric growth patterns among strains. A repeated measures analysis was conducted on the first six relative warp scores with the average family scores used as the dependent variables to determine if strain differences in morphological change existed over time. Mixed model analysis with strain and maturation status as fixed effects, and sire and dam as random effects were constructed for each time period to test whether there were significant differences among strains for relative warps one through six, length, weight, relative condition factor, and development rate; Tukey's post-hoc test was used to test for significant differences between strains. Mixed model analysis with dam as random effect was used to test for significant differences in egg diameter among strains. A sub-sample of an equal number of individuals from each family was used in a discriminant function analysis (DFA) with the observation being classified left out. The DFA was conducted on partial warp scores, to determine whether differences observed between strains were powerful enough to reclassify according to strain. Tests for departures from normality were conducted for all traits prior to the above analyses. All statistical analyses were conducted using SAS 9.1 statistical software (SAS; Cary, North Carolina).

#### Heritability estimates

Variance components used to estimate heritability were calculated with a mixed model method, the animal model, using ASREML software [67]. The following univariate animal model was used to calculate variance components
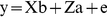
(3)where y is the vector of relative warp scores, length, or weight, b is the vector of fixed effects, a is the vector of random additive genetic effects, X and Z are the corresponding design matrices which relates the effects of Y, and e is the vector of residual values [49], [68]. The population means were fixed effects and individuals were random effects; the genetic variance-covariance among individuals is a function of the additive genetic relationships between individuals and the variance in additive genetic effects (V_a_), which is then used to calculate heritability of individual traits for each strain [49], [68]. During sampling periods two and three, a number of males had matured; therefore, maturation status was used as an additional fixed effect for those sampling periods. Strain was used as an additional fixed effect when determining overall heritability for all strains together. Heritability (h^2^) was calculated as the ratio of additive genetic variance (V_a_) to total phenotypic variance (V_p_). To test the hypothesis that heritability is significantly different from zero (V_a_>0), a likelihood-ratio test was performed as twice the difference in log-likelihoods between the full model mentioned above and the reduced model without random animal effects. The P values were approximated from a χ^2^ distribution with one degree of freedom. The following model was used to test for dam effects
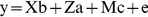
(4)where y, Z, a, and e are as stated above, c is the vector of random dam effects, and M is the corresponding design matrix. Dam effects were calculated as the proportion of total phenotypic variance due to dams and may consist of maternal environment, other common environmental effects encountered before families were tagged, or dominance deviation effects. Significance was tested with a likelihood-ratio test of the full model and the reduced model excluding random dam effects and P values were approximated as described above. When significant dam effects were detected, random dam effects were included in the model to determine heritability. Finally, family effects were included as an additional random effect in models 3 and 4 above, and the likelihood ratio test was used to test whether common environment shared by members of the same family was a significant contributor to variation in phenotype. Family effects included in the models were intended to account for environmental differences among families, including but not limited to slight differences in tank and family densities, and any effects caused by the sharing of buckets during early rearing. Genetic covariances and correlations were calculated in the context of the same animal model, but using multivariate analyses of two traits at a time. To test the hypothesis that individual genetic correlations were zero, models constraining genetic covariance to zero were compared to full models without such constraints in likelihood ratio tests.

## Results

### Length, weight, and condition factor

**Figure 2:**
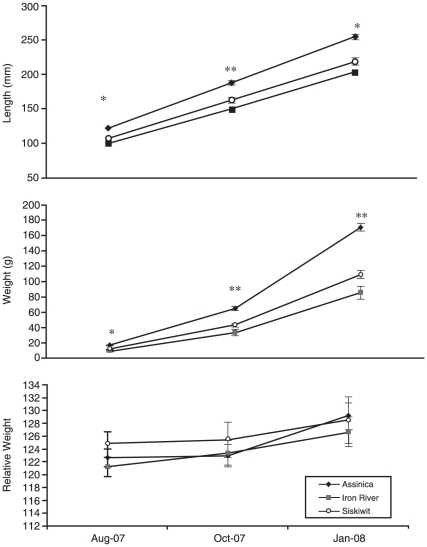
Length, weight, and relative condition measurements of brook trout strains at each sampling period with standard error bars. * indicates a significant (P<0.05) difference between all three brook trout strains, ** indicates Assinica strain is significantly different from both Siskiwit and Iron River.

Significant differences among strains were found in length and weight at multiple sampling periods. Assinica individuals were the largest and Iron River individuals were the smallest in both length and weight throughout the experiment (Figure 2). All strains were significantly different in length at sampling period one (all P<0.05) and three (all P<0.05); significant differences were found between Assinica and the other two strains at sampling period two (all P<0.0001), while Siskiwit and Iron River strains were not significantly different (P = 0.0871). All strains were significantly different in weight at the first sampling period (all P<0.05). Assinica individuals were significantly different from Iron River and Siskiwit in weight measurements for the second (all P<0.0001) and third (all P<0.0001) sampling periods. Siskiwit and Iron River individuals were not significantly different in weight at the second (P = 0.1229) and third (P = 0.0644) sampling period. No significant differences in relative condition factor were found among strains at any sampling period (data not shown). No significant differences were found among strains in egg diameter or development rate (data not shown).

### Morphometrics

**Figure 3:**
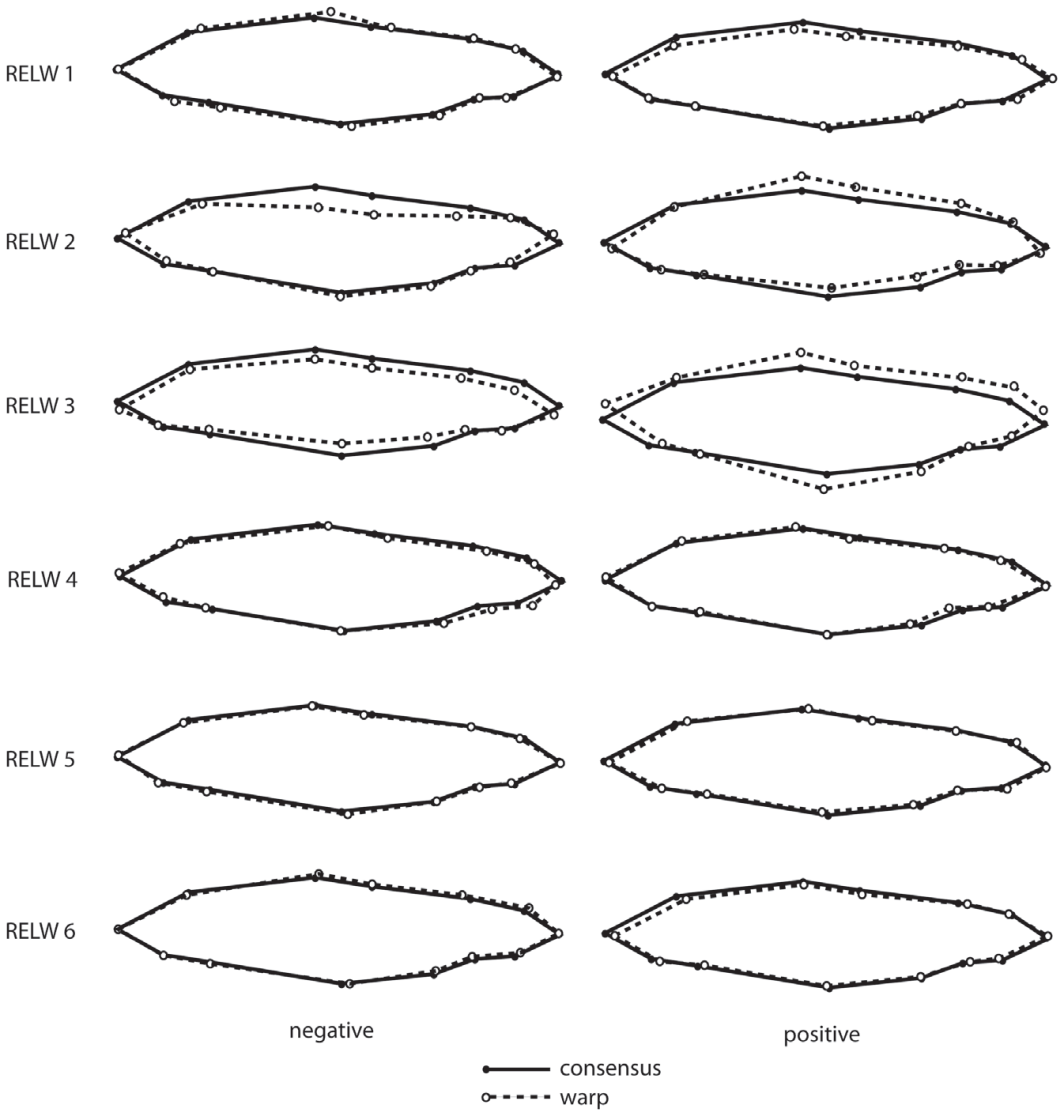
Relative warps from geometric morphometric analysis of body shape. Positive and negative most extreme relative warps (dashed lines) are compared with the consensus shape (solid lines) for relative warps one through six, with the left side of the fish pictured.

**Figure 4:**
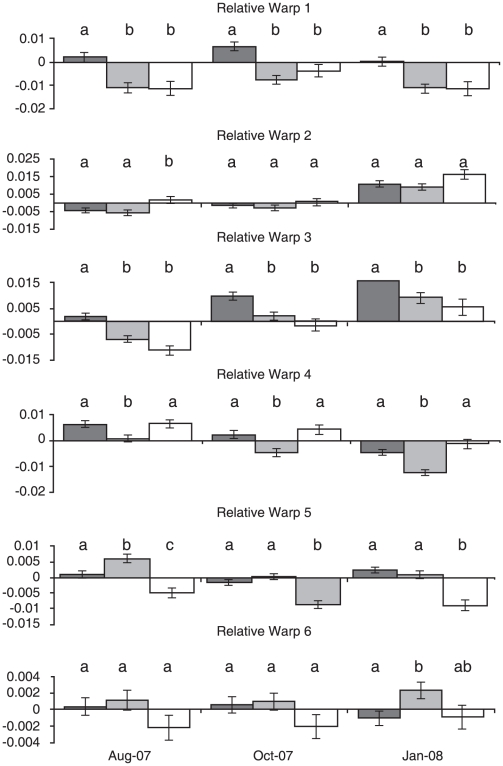
Least squares means with standard error bars of relative warps one through six for Assinica (dark gray), Iron River (light gray), and Siskiwit (unfilled) brook trout. Letters denote Tukey groupings (P<0.05) of strains within each of the three sampling periods.

Relative warp analysis resulted in 22 warps. The first six relative warps explained 74.51% of total variation in body shape and were chosen for further analysis and discussion. The first relative warp explains 23.58% of the total variation and describes the length of the head, placement of the dorsal and pelvic fin, body depth at the midsection, and caudal peduncle region. Positive scores indicate specimens with shorter heads, more anterior placed dorsal and pelvic fins in relation to the posterior end of the body, slimmer body depths at the midsection, and longer caudal peduncles; negative scores indicate the opposite form (Figure 3). Assinica had significantly higher scores than both Iron River and Siskiwit at all three sampled time points (all P<0.01; all P<0.05; all P<0.01) (Figure 4). Warp two explains 18.21% of total variation and is dominated by body curvature and variation in the shape of the head. Positively scored individuals had a concave curvature, larger mouths, and a more anterior position of the back of the head (Figure 3). Siskiwit had significantly higher scores than both Iron River (P = 0.0168) and Assinica (P = 0.0485) during sampling period one; however, no significant differences were found during sampling periods two and three (Figure 4). Warp three explains 13.22% of variation and describes variation in the shape of the head, body depth, and caudal peduncle depth. Positively scored individuals were deeper across the entire body length, including the caudal peduncle, have longer premaxillary lengths and upturned snouts. Negatively scored individuals were very slender with smaller down turned mouths (Figure 3). Assinica had significantly higher warp scores than Iron River and Siskiwit during the first sampling period (Iron River: P = 0.004; Siskiwit: P<0.0001), second (Iron River: P = 0.0019; Siskiwit: P = 0.0003), and third (Iron River: P = 0.0012; Siskiwit: P = 0.0005) (Figure 4). Siskiwit and Iron River were not significantly different for warp three at all sampling periods (P = 0.1431; P = 0.3038; P = 0.4279). Warp four explains 8.30% of total variation; the head was larger and the dorsal fin was more anterior in relation to the pelvic fin in positively scored individuals and the area between the caudal peduncle and the end of the body was larger indicating a larger region of caudal fin attachment (Figure 3). Iron River had significantly lower scores for relative warp four than Assinica and Siskiwit at all three time points (all P<0.05; all P<0.01; all P<0.0001) (Figure 4). Relative warp five explains 6.86% of total variation. Positively scored individuals were slender bodied in the area between the dorsal and pelvic fins created by an anterior shift in the pelvic fin. Positively scored individuals also have longer caudal peduncles, longer dorsal fins, and more posterior dorsal fins. Negative individuals were opposite in form (Figure 3). During sampling period one, all strains were significantly different from each other for relative warp five (all P<0.05), with Iron River having the highest scores and Siskiwit having the lowest. During sampling period two (Iron River: P<0.0001; Assinica: P = 0.0003) and three (Iron River: P = 0.0001; Assinica: P<0.0001), Siskiwit individuals had significantly lower scores than Assinica and Iron River (Figure 4). Assinica and Iron River individuals were not significantly different in warp five during the second (P = 0.3150) and third (P = 0.5207) sampling periods. Relative warp six explains 4.35% of total variation. Positively scored individuals had shorter caudal peduncles, longer anal fins, more anterior placed dorsal and pelvic fins in relation to the caudal peduncle, and a slender body in the mid and posterior sections (Figure 3). At sampling period three, Assinica fish had significantly lower values for relative warp six than Iron River (P = 0.0357), while Siskiwit individuals were not significantly different than Iron River (P = 0.1649) or Assinica (P = 0.9967) individuals. No significant differences were found among strains during periods one and two (Figure 4). All other relative warps explained less than 4% of the variation in morphology.

Morphometric characters showed no obvious departures from normality. The MANOVA of partial warp scores yielded significant results in body morphology among all three strains of brook trout throughout the experiment (Wilks' Λ = 0.8204, P<0.0001; Wilks' Λ = 0.8326, P = 0.0001; Wilks' Λ = 0.7710, P = 0.0002; sampling periods one, two, and three, respectively). MANCOVA results of interactions between log_10_ centroid and strain reveal significant interactions for all sampling periods (Wilks' Λ = 0.8174, P<0.0001; Wilks' Λ = 0.8395, P = 0.0003; Wilks' Λ = 0.8010, P = 0.0036) indicating a difference in the effect of size on morphology among strains. Discriminant function analysis yielded correct classification rates of individuals to strains of 75.28% during sampling period one, 76.60% during sampling period two, and 73.54% during sampling period three (Table 1). The repeated measures analysis revealed significant strain by time interaction effects for relative warp four (P = 0.0072) and relative warp five (P<0.001), indicating that the change in these morphological metrics are different for each strain over time. Time effects were significant for relative warp one (P<0.0001), relative warp two (P<0.0001), and relative warp three (P<0.0001), indicating significant changes in morphology over time.

**Table 1:** Discriminant function analysis cross-validation reclassification percentages for brook trout strains at three sampling periods.

	Aug-07			Oct-07			Jan-08		
Original strain	Assinica	Iron River	Siskiwit	Assinica	Iron River	Siskiwit	Assinica	Iron River	Siskiwit
Assinica	**75.24**	17.14	7.62	**73.85**	15.38	10.77	**74.42**	11.63	13.95
Iron River	5.26	**78.95**	15.70	7.02	**85.96**	7.02	21.05	**68.42**	10.53
Siskiwit	15.00	13.33	**71.67**	16.67	13.33	**70.00**	11.11	11.11	**77.78**

### Heritability

Most relative warps, length and weight had significant, non-zero heritability estimates in all three strains of brook trout, with several values being high (Table 2). All estimates of heritability for length were significant and ranged from 0.23–0.93; significant heritabilities for weight ranged from 0.32–0.88, with weight of Iron River during sampling period three being the only non-significant result. Heritability for all six relative warps through time was significant in the Assinica strain (0.22–0.95). Relative warps three through six showed non-zero heritabilities through time in Iron River (0.24–0.81), warp one was not significant at any time period and warp two only during sampling period two. In Siskiwit, heritability of all relative warps were significant during sampling period one (0.19–0.86); warps one, two, and six were significant for sampling period two and no warps had significant heritabilities during sampling period three. Only two instances of significant dam effects (m^2^ = V_m_/V_p_, where V_m_ is variance due to dam effects) were found for all traits measured; during sampling period three, Assinica relative warp five (m^2^ = 0.25±0.12, P = 0.0421) and during sampling period one, Iron River relative warp three (m^2^ = 0.25±0.09, P = 0.0218) were significant. Common environment shared by families was not significant for most traits and time points within strains, and exceptions are noted in Table 2. Heritabilities calculated with family included in the models for which it was significant were not significantly different from zero in formal likelihood ratio tests, even though some standard errors for estimates of heritability do not overlap with zero; this result likely stems from the very low amount of power to separate family and additive genetic effects due to the small number of families available. Family effects were completely confounded with the additive genetic relationships in the Siskiwit crosses, as female egg lots were not split amongst males, and thus family effects could not be estimated separately. When considering all strains together, accounting for strains as a fixed effect, significant heritability estimates were found for all relative warps at all sampling periods (Table 3). Finally, though genetic correlations were calculated between traits in the context of a multivariate animal model, standard errors were large and formal tests to determine if correlations were different from zero failed to detect significant correlations both within individual strains, and across all strains combined (data not shown).

**Table 2:** Narrow-sense heritability estimates for morphometric characters described in terms of relative warps (RW), length and weight.

	August 2007			October 2007			January 2008		
	Assinica	Iron River	Siskiwit	Assinica	Iron River	Siskiwit	Assinica	Iron River	Siskiwit
RW1	0.90±0.19**	0.08±0.07	0.81±0.26**	0.92±0.20**	0.00	0.48±0.29**	0.78±0.20**	0.04±0.11	0.83±0.44
RW2	0.22±0.12**	0.10±0.08	0.19±0.15*	0.45±0.17**	0.50±0.19**	0.47±0.29**	0.27±0.15**	0.14±0.14	0.00
RW3	0.66±0.18**	0.60±0.18**	0.34±0.20**	0.90±0.20**0.55±0.35^a^	0.81±0.21**	0.14±0.23	0.77±0.22**	0.24±0.17*	0.00
RW4	0.86±0.19**0.56±0.29^a^	0.75±0.20**	0.64±0.25**	0.73±0.20**	0.66±0.21**	0.51±0.34	0.34±0.19**	0.39±0.21**	0.60±0.50
RW5	0.72±0.19**	0.60±0.18**	0.36±0.21**	0.78±0.20**	0.52±0.20**	0.33±0.32	0.71±0.22**	0.42±0.20**	0.20±0.38
RW6	0.95±0.19**0.47±0.46^a^	0.48±0.17**0.09±0.30^a^	0.86±0.26**	0.62±0.19**	0.40±0.17**	0.65±0.33**	0.68±0.21**	0.43±0.22**	0.81±0.46
Length	0.44±0.16**0.02±0.25^a^	0.50±0.18**	0.71±0.25**	0.57±0.19**	0.53±0.20**0^a^	0.73±0.31**	0.50±0.20**	0.23±0.17*	0.93±0.38**
Weight	0.32±0.14**0^a^	0.49±0.18**	0.80±0.26**	0.52±0.18**0.28±0.27^a^	0.62±0.21**0^a^	0.60±0.30**	0.38±0.17**	0.10±0.13	0.88±0.38**

*significance at 0.05,

**significance at 0.01.

a In cases where two heritabilities are reported, the bottom value is heritability with family included in the model.

**Table 3:** Narrow sense heritability estimates for morphometric characters described in terms of relative warps across all three strains of brook trout.

Sampling Period	Relative Warp 1	Relative Warp 2	Relative Warp 3	Relative Warp 4	Relative Warp 5	Relative Warp 6
1	0.65±0.12	0.16±0.06	0.59±0.11	0.77±0.12	0.60±0.11	0.79±0.12
2	0.61±0.13	0.45±0.12	0.72±0.13	0.68±0.13	0.64±0.13	0.55±0.12
3	0.39±0.13	0.21±0.10	0.55±0.15	0.37±0.12	0.53±0.14	0.67±0.15

All values are significant at p<0.001.

## Discussion

The degree of additive genetic variance for quantitative traits has important implications for the evolutionary trajectories of both natural and captive populations. The results of this study indicate that there is significant additive genetic variation for morphology and body size within and across all three brook trout strains examined. Morphology and body size can be influenced by environmental conditions but our findings indicate that genetic variation also contributes to this phenotypic variation. The large levels of additive genetic variation suggest that natural or artificial selection for a single or optimal morphotype is not strong within these strains, and it is possible that heterogeneity in the natural or hatchery environments (microhabitat, density, food availability, temperature) has selected for and maintained variable morphotypes. Moreover, body morphology bears significant additive genetic variation in these brook trout strains, indicating that the hatchery populations are capable of responding to selection in both natural and artificial environments.

The empirical estimates of heritability of morphological, weight, and length characters in this study are similar to values observed for other species of salmonid fishes. The median value for point estimates of heritability for morphometric characters, length, and weight was 0.52 in this study, a value higher than the median value of 0.29 observed from a meta-analysis of heritability for all morphological characters (including morphometric characters, length, weight) across salmonid species [41]. Only 3% of the heritability values used for meta-analysis in Carlson and Seamons [41] were conducted in brook trout, illustrating that very little data on quantitative genetic parameters have been published for this species when compiled with all other salmonid species. Though there are a number of studies that estimate heritability for a number of brook trout characters (see Carlson and Seamons [41] for a review), to date there is only a single other study that formally evaluates the additive genetic variation for body shape in this species [69]. In sympatric anadromous and resident brook trout in a single river system, Theriault et al. [69] found that fork length had a significant heritability of 0.50, compared to a range of 0.23–0.93 for length in our study. Morphological features were analyzed in different ways in the two studies. For the morphological features measured, Theriault et al. [69] found significant heritabilities only for pelvic fin length (0.40) and maximum body width (0.28); notably, peduncle depth heritability was not significant in Theriault et al. [69], but relative warps that explained variation in this feature were significantly heritable in our study. Again, caution is taken in comparing results across studies, as characters may be measured using different methods, and in different environments, which are known to influence both the expression of additive genetic and phenotypic variation within and among studies.

The variable morphologies found in our sample could have performance consequences for these populations in both the hatchery and natural environments. Assinica brook trout (originating from a migratory natural population) were consistently different from both Iron River and Siskiwit strains within and among sampling periods; on average Assinica had deeper bodies and caudal peduncles, long caudal peduncles, short heads, dorsal fins placed in a more anterior position, and more dorsally oriented mouths than fluvial Iron River and migratory Siskiwit strains. The deep body and more dorsally oriented mouth of Assinica are well suited for burst swimming, quick turns and may be associated with increased prey capture success in drift feeding situations [70]. However, the morphology observed in the Assinica strain would be expected to be found in fluvial not migratory populations, and may likely also be related to inadvertent artificial selection during its long time in the hatchery. Fluvial Iron River brook trout had an intermediate body depth and mouth position relative to Assinica and Siskiwit. In addition, Iron River fish had a short caudal peduncle and short heads. Migratory Siskiwit brook trout were the most slender with the most ventrally oriented mouths of the three strains and had a shorter posterior-placed dorsal fin, more posterior pelvic and anal fin placement, and short caudal peduncles. The streamlined bodies of Siskiwit brook trout along with small dorsal fins reduce drag and are beneficial in cruising and sustained swimming situations and would be expected in migratory ecotypes [20], [71]. The Siskiwit body morphology suggests juveniles are better adapted to open water or swift currents and may occupy areas of fast current in stream environments to increase prey capture efficiency [20], [70]. Morinville and Rasmussen [4], [72] found significant differences in morphology and metabolic costs between resident and anadromous brook trout. Anadromous individuals consumed more and had lower growth efficiencies in which differences may be attributed to standard metabolic rate or activity. Individuals with high activity levels due to lower rates of prey capture or predator avoidance may be less adapted to their environment and would benefit from a change in location. Lower fitness potential experienced in the stream environment and heritable differences in morphology may lead to and sustain a migratory tactic. A lower fitness may also be observed if brook trout of certain morphologies are stocked in streams with unsuitable habitat rather than habitat more conducive to their morphology. Relative condition factor was also measured as it can indicate differences in form. No differences in relative condition factor were found among the three strains indicating differences in length and weight did not lead to differences in plumpness of the fish.

Another source of differentiation among these hatchery strains may be length of time in hatchery. The hatchery setting may induce selection of morphologies that differ from that in the wild. Some studies have found hatchery juvenile coho and chinook salmon to have smaller heads, smaller median fins, and more slender bodies than wild fish [29], [30], [73]; Fleming and Gross [28] also found adult female coho of hatchery origin to be more streamlined with smaller fins. Differences between hatchery and wild populations are not always found; Dahl et al. [74] found no significant differences between wild and hatchery brown trout (*Salmo trutta*); however, hatchery conditions and length of time the broodstock has been maintained in hatcheries may have an effect on these variables. Assinica brook trout have been domesticated for the longest time (over 45 years) yet had the deepest bodies. Currently Assinica and Iron River strains are held in the same hatchery under similar conditions however significant differences are still found between the strains; differences among these strains could be a product of not only ecotypic diversity in the source populations, but also the length of time in the hatchery system and exposure to artificial selection for morphology. Siskiwit brook trout have been domesticated for the shortest amount of time but had the most slender body shape of the three strains. The similarity of these hatchery strains to their source populations would be an interesting future study, both to compare the extant quantitative genetic diversity in both source and hatchery populations, and to examine morphological differentiation within and among natural populations.

Discriminant function analysis yielded re-classification rates ranging from 68% to 86%, depending on strain and sampling period. Common rearing environments and family structure may have lead to more similar morphologies than would be expected in the wild due to differences in habitat selection and behavior; however, these classification rates, on the upper bound, are similar to that observed among life history tactics in a single population of brook trout reared in their natural environment, where the overall classification rate was 87% [4].

The absence of significant heritability in some samples may be a function of small sample sizes within families or a small number of families and not an absence of genetic variation. An illustration of this may be found both in the progressively low sample sizes for Siskiwit during each developmental time point, and in the large estimates of standard errors in the later time points. The sample size (and number of families) for Siskiwit during sampling period two was small (65 individuals) and even smaller during sampling period three (35 individuals) and may have had an effect on our ability to detect heritable morphologies. Heritabilities of Assinica strain were consistently found to be significant, they also had the greatest number of families for the detection and quantification of heritability.

In the case of Assinica brook trout, all warps were found to be heritable. The Assinica strain went through a serious bottleneck when founded, as only four females and three males were used as founders. The Siskiwit strain was also founded from a small number of individuals. Heritability of morphological and life history traits have been found to increase immediately after population bottlenecks due to non-additive variance such as epistatic and dominance effects [75], [76]. Additive genetic variance for life history traits is highest at intermediate inbreeding coefficients. If the hatchery environment provides limited selection, additive genetic variance would be expected to remain high after a bottleneck and may explain the inflated additive variance found in the Assinica strain. In contrast the Siskiwit hatchery strain was started with a number of outbred, wild individuals in 2004. Outbred populations can show significantly lower additive genetic variation in life history traits than inbred populations [76]. The U.S. Fish and Wildlife Service has maintained strict breeding methods since a broodstock was developed from the Siskiwit population and these methods were analyzed by Cooper et al. [77] through molecular genetic diversity measurements. Cooper et al. [77] found levels of genetic diversity within the Siskiwit hatchery population to be consistent with that observed in the wild population.

Heritability can change over the course of ontogeny as a result of variable environmental contributions, directional or stabilizing selection, compounding of genetic effects, or canalization [78], [79]. In our study, most of the heritabilities across time points for individual traits within strains remained within one standard error. In a few cases, traits that had significant additive genetic variation at one time point had non-significant additive genetic variation at another time point. The inability to detect additive genetic variation for some traits at some time points is more likely attributed to low samples sizes (and power). Though the number of families remained the same between time points (with the exception of a single Siskiwit family in the final time period), the within-family sample sizes decreased which may have had an impact on our ability to detect significant genetic variation for traits at the later sampling periods. Maternal effects were not significant for most traits at all developmental time points in our study; it appears that maternal effects that are prominent during embryonic development [80] have largely disappeared within the first year for body size and shape within these populations. This is consistent with the observation that maternal effects decline with age in domesticated animals [81], and with the observation that maternal effects were not observed in brook trout for traits measured after the swim-up stage [82]. Though common rearing environment can cause an upward bias in the estimation of heritability, we observed significant family effects (due to common environment) in a minority of traits; however, it should be noted that due to limited space and gametes for families, our breeding design did not have a lot of power to detect these effects, or to estimate heritability while accounting for common environmental effects.

Morphology is also expected to change over ontogeny. Studies have shown morphological differences during out-migration between migratory and resident brook trout [4]. Some Lake Superior brook trout may emigrate from natal streams as early as age 1+ [60], therefore morphological adaptations to migration are likely to occur before this age. In fact, Perry et al. [80] found differences in size between anadromous and resident brook trout as early as the embryo stage, and Chernoff and Curry [83] found size differences during the first three months post-emergence. However, migration in some fish or populations may not occur until age 2+ [84], in this case morphological adaptations to the lake environment may not appear until later life stages in which this study did not cover.

In summary, our study suggests that differences in morphology exist between hatchery brook trout strains with variable life histories and that there is significant genetic variation for size and morphology within and among these populations. Significant, non-zero additive genetic variance and heritabilities indicate an ability to adapt and a genetic contribution to morphology which will prove to be an important factor in the maintenance and evolution of life history variation through both natural processes and in attempts to restore natural populations of brook trout. We cannot separate the confounded effects of life history origin and hatchery or domestication effects in this study; however, we have demonstrated that strains currently and historically used for stocking and supplementation do have significant quantitative genetic variation for morphology, which may have performance consequences in the natural environment and can shape the evolutionary trajectories of populations derived from these hatchery strains. The question of whether the levels of additive genetic variation in this study reflect that found in the source populations from which they were derived can only be answered in a study evaluating heritability in the natural populations and environment. Evaluating heritability of morphology, behavior, physiology and life history in wild populations of both pristine and imperiled brook trout populations is an important next step in understanding the degree to which life history variation is influenced by underlying genetic variation, and the amount of genetic variation available for the evolution of these traits.
